# Supplementary figures and images for: The hGIDGID4 E3 ubiquitin ligase complex targets ARHGAP11A to regulate cell migration
Source: Life Sci Alliance. 2024 Oct 10;7(12):e202403046. doi: 10.26508/lsa.202403046 (PMC11467045; doi:10.26508/lsa.202403046)

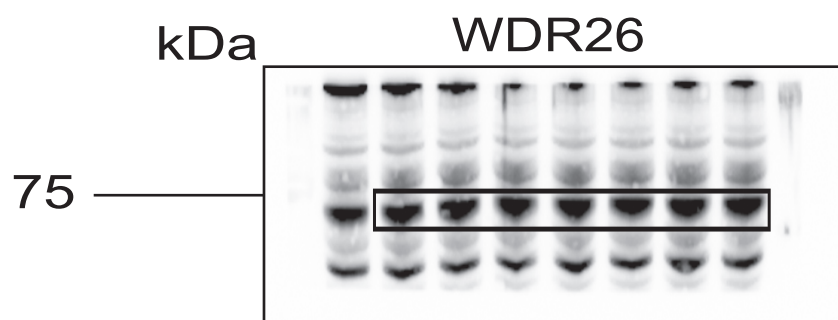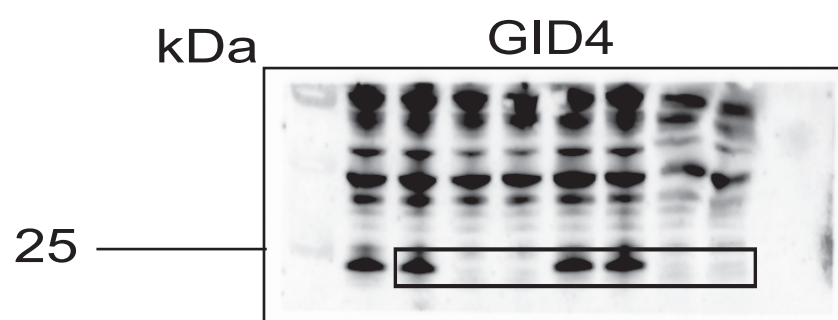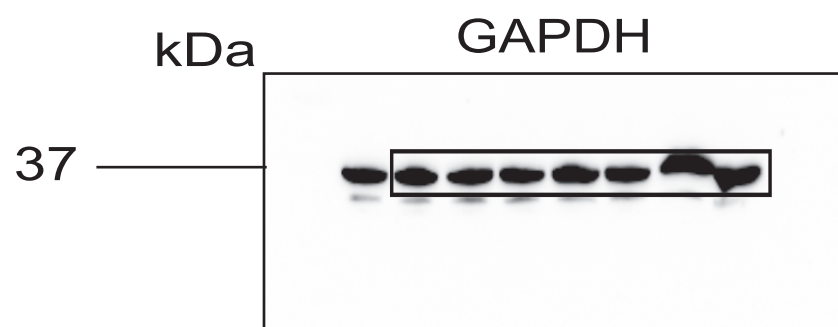

Supplement: Supplementary file 1 [file LSA-2024-03046_SdataF1.pdf]

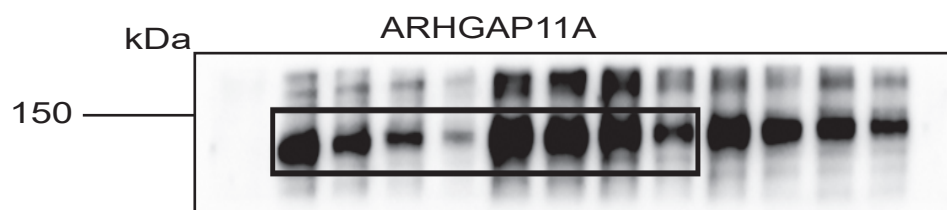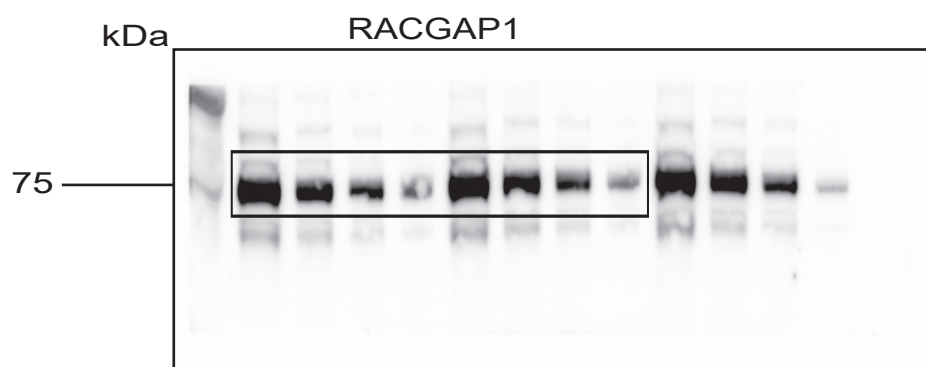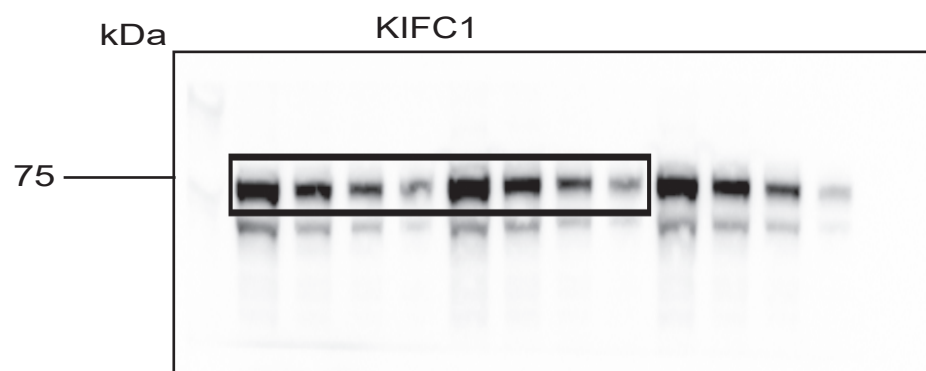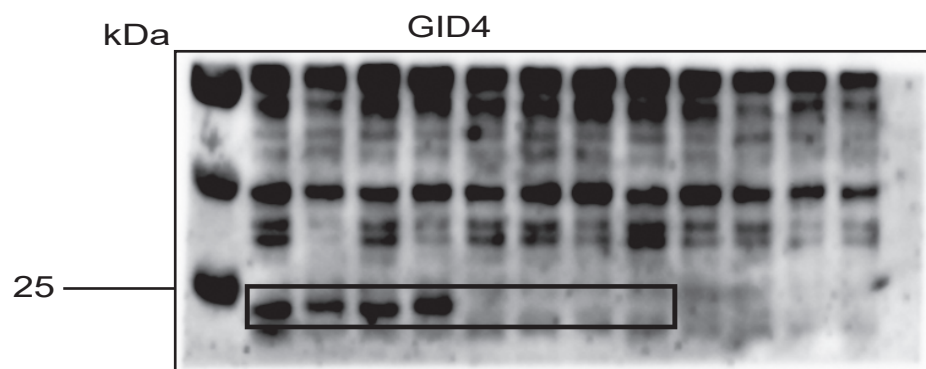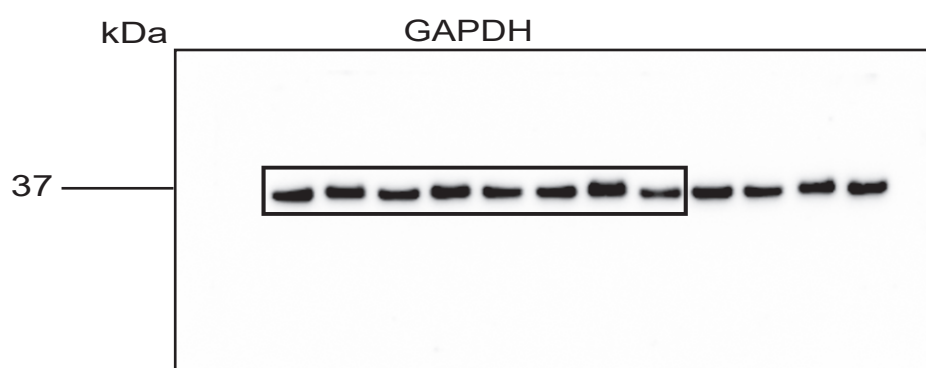

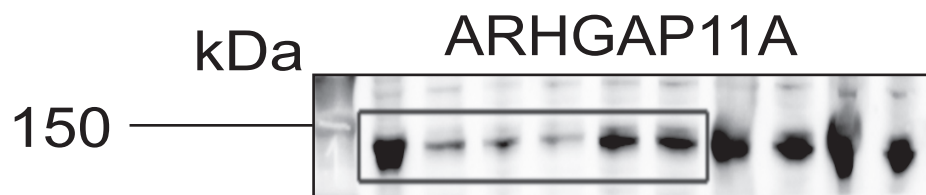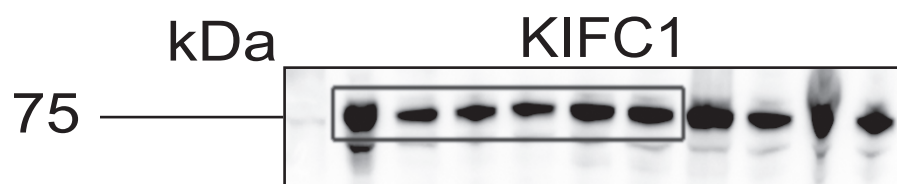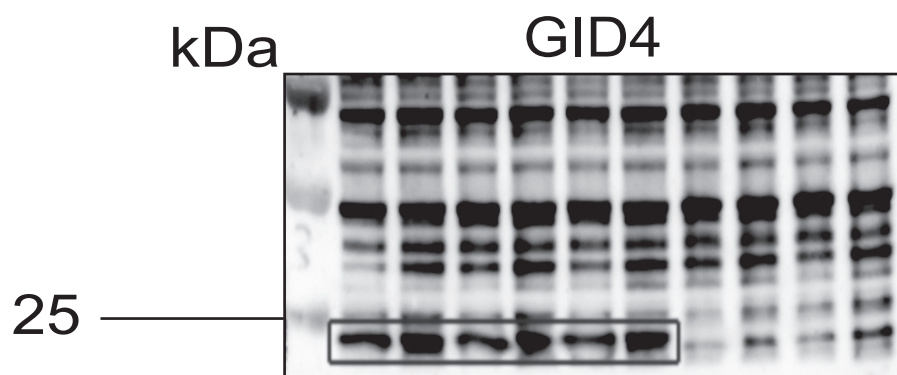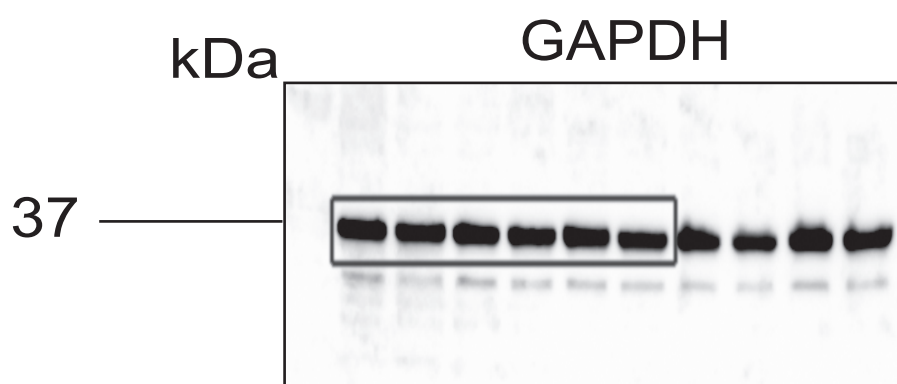

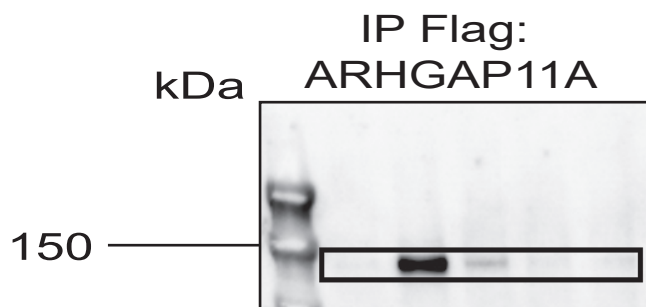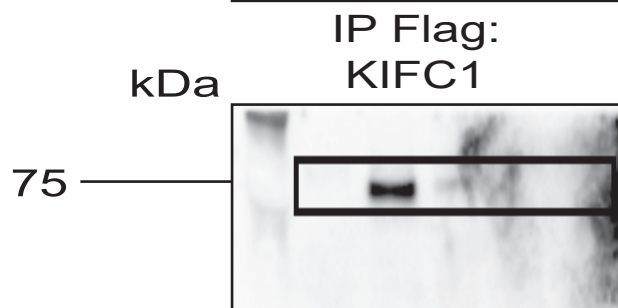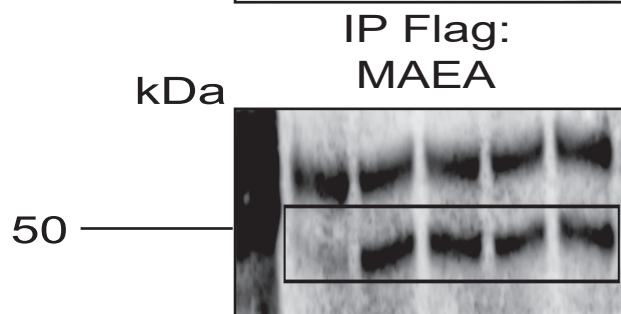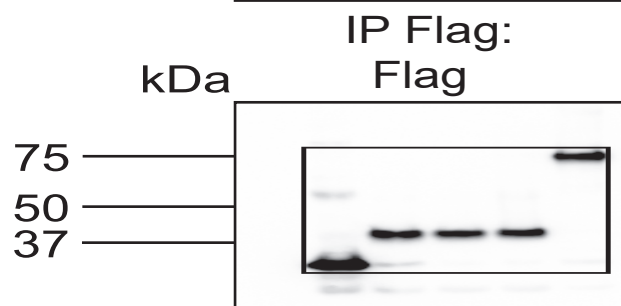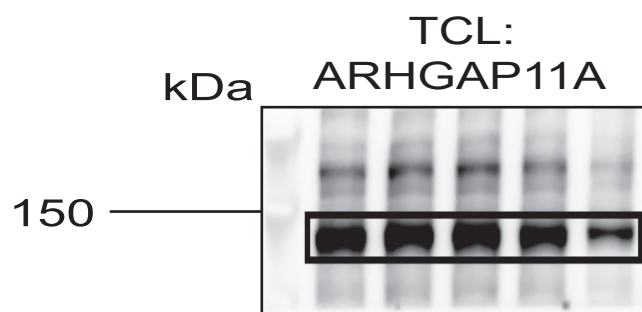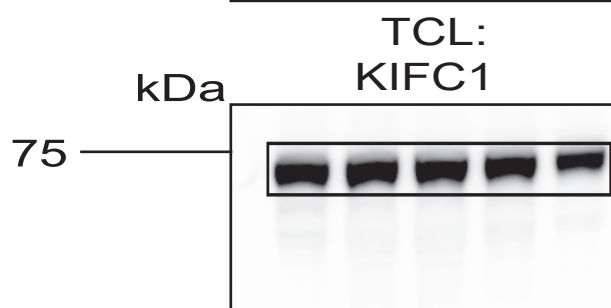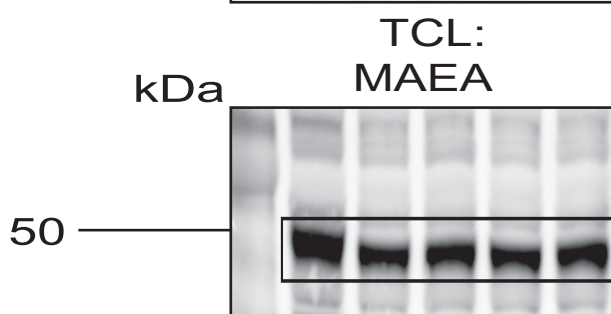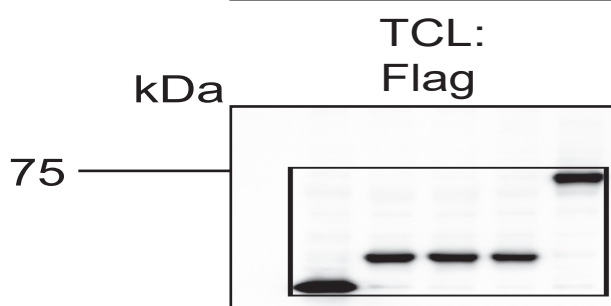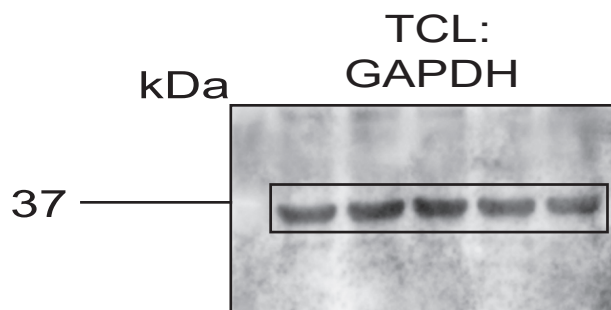

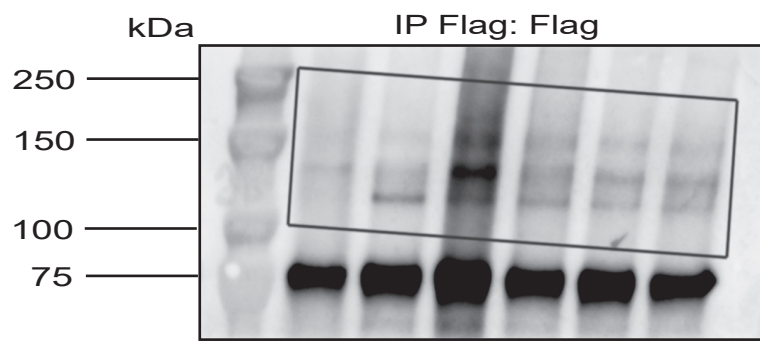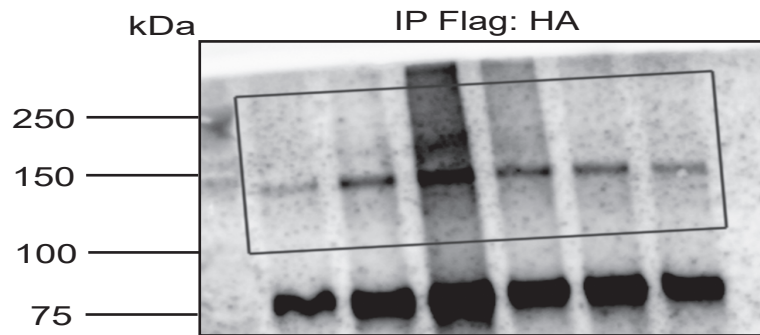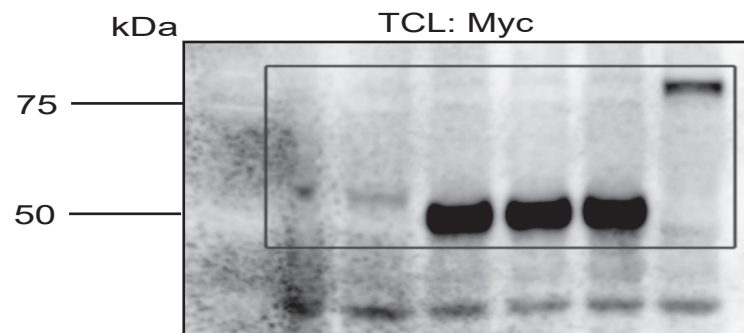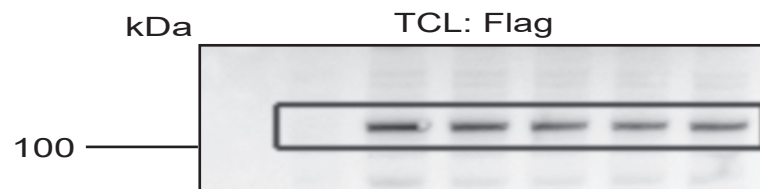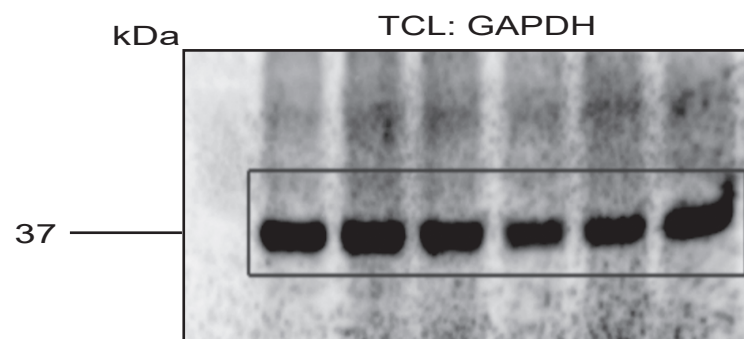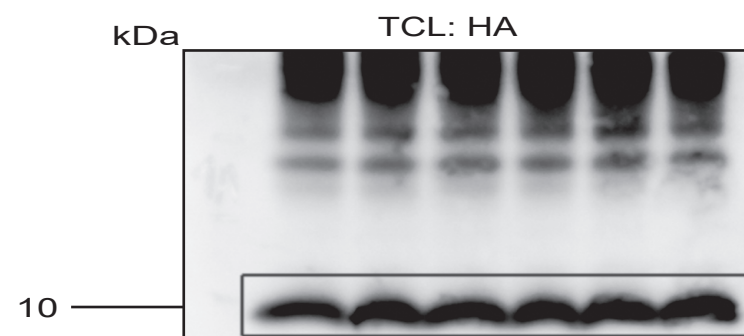

Supplement: Supplementary file 5 [file LSA-2024-03046_SdataF3.pdf]

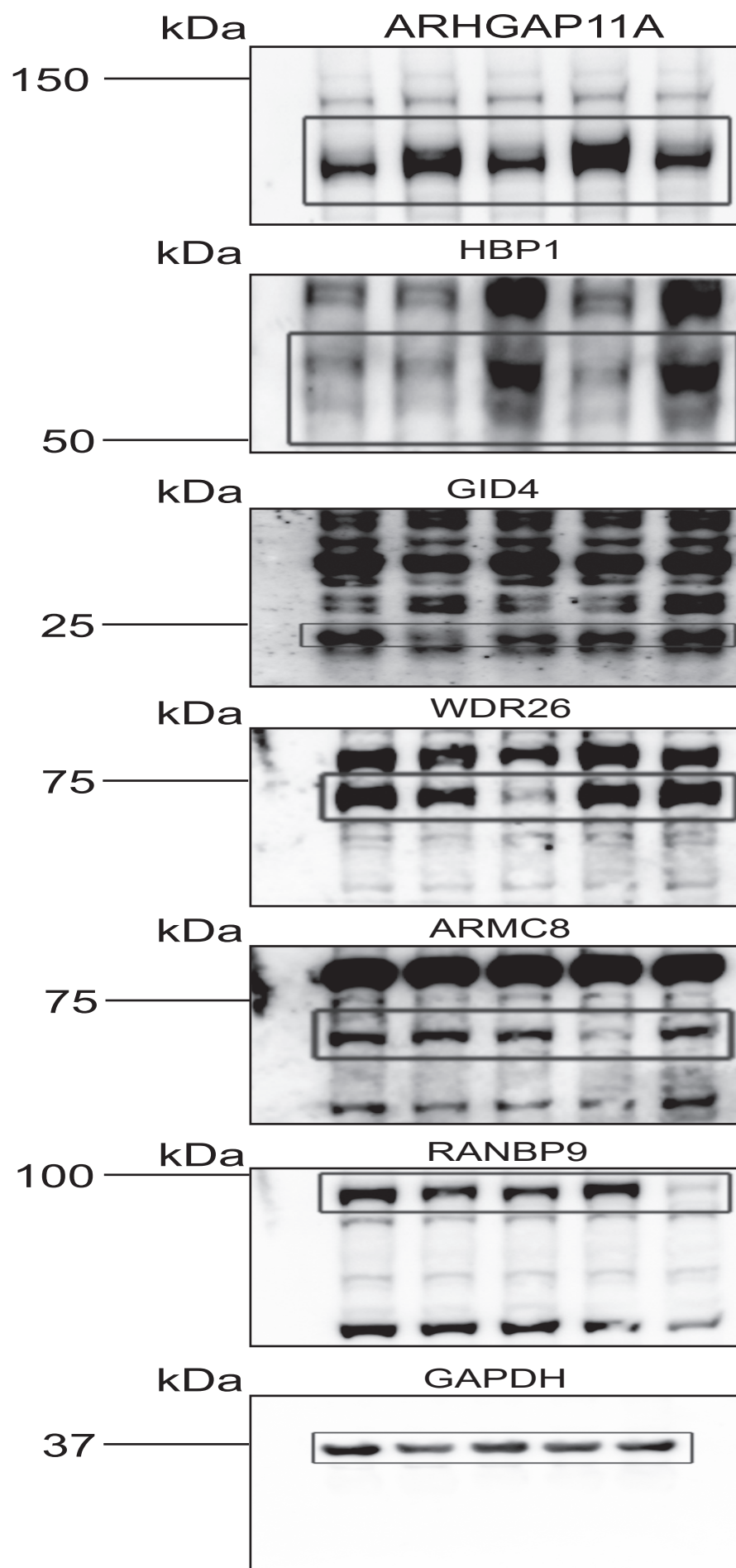

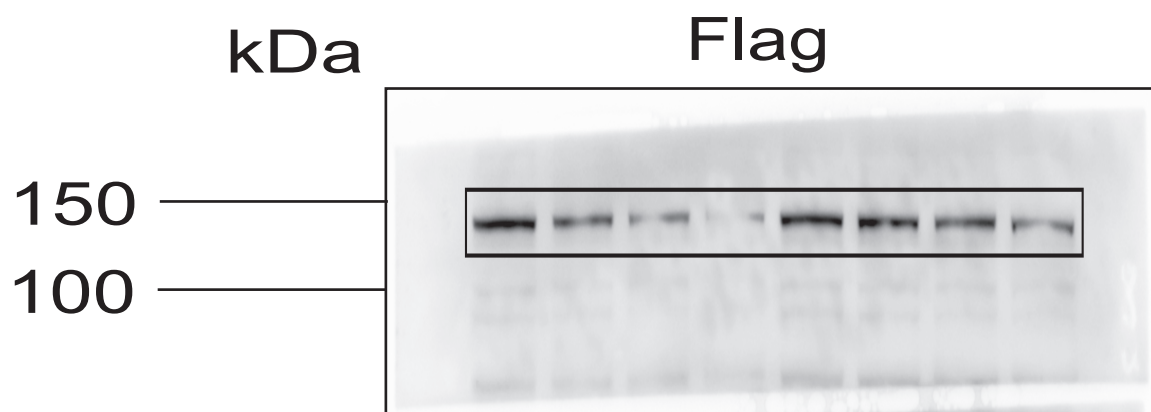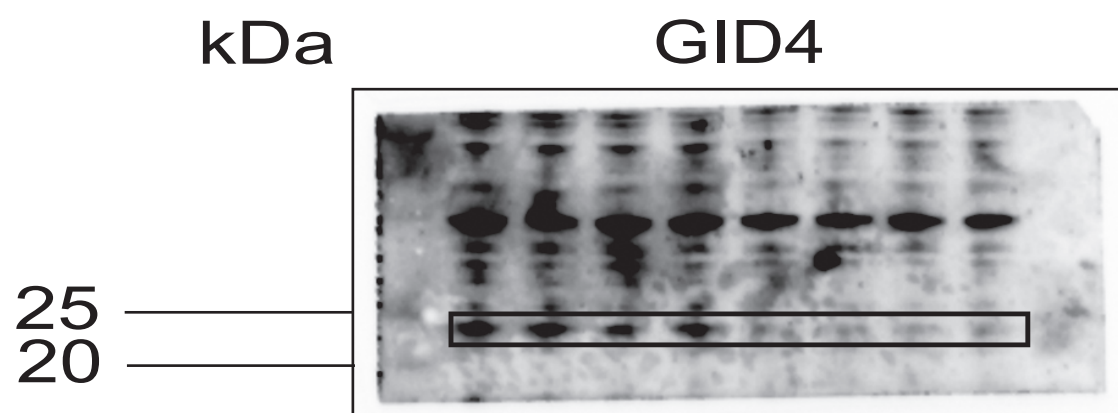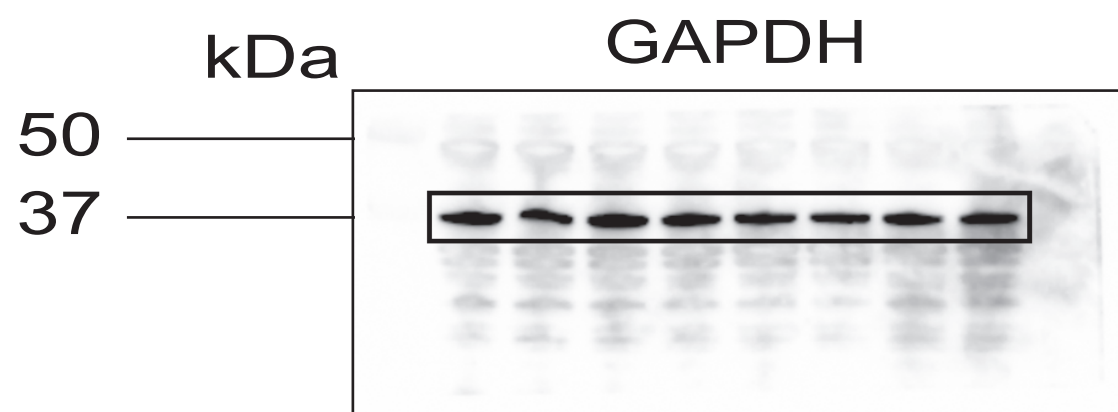

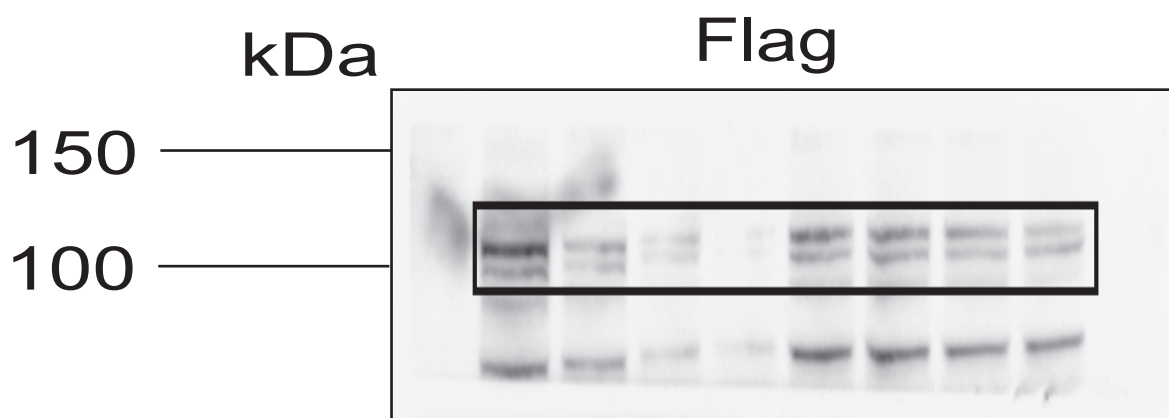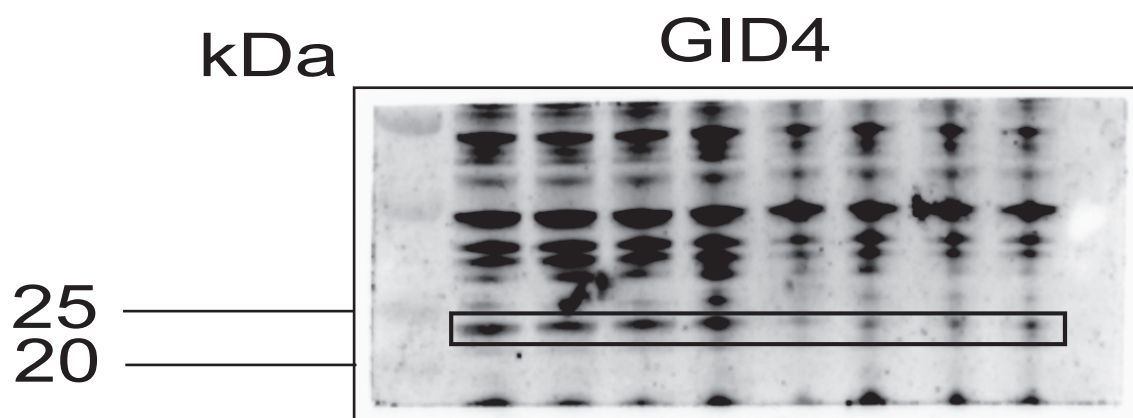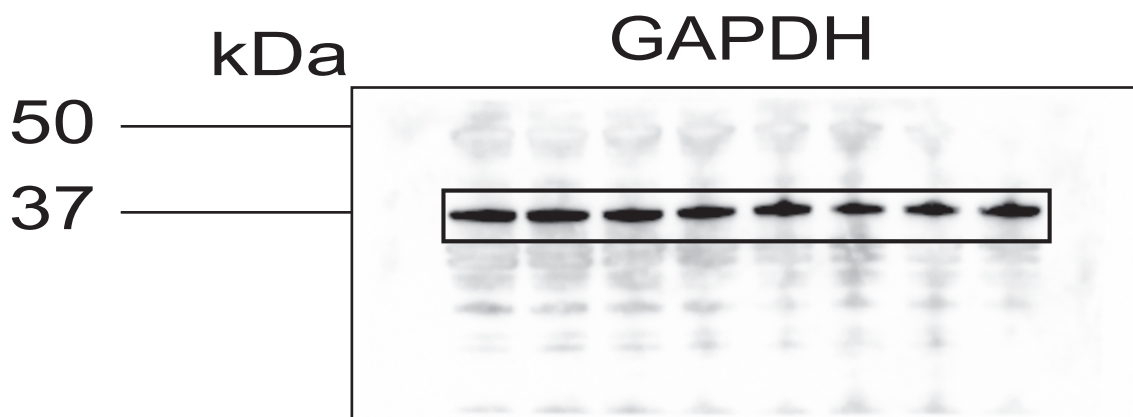

Supplement: Supplementary file 6 [file LSA-2024-03046_SdataF4.pdf]

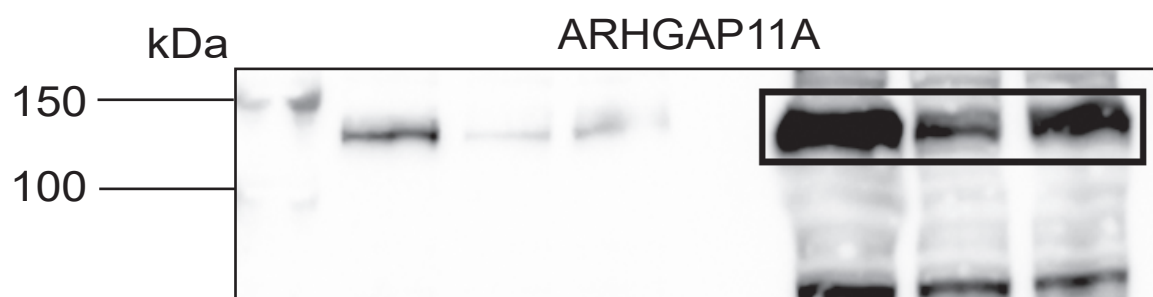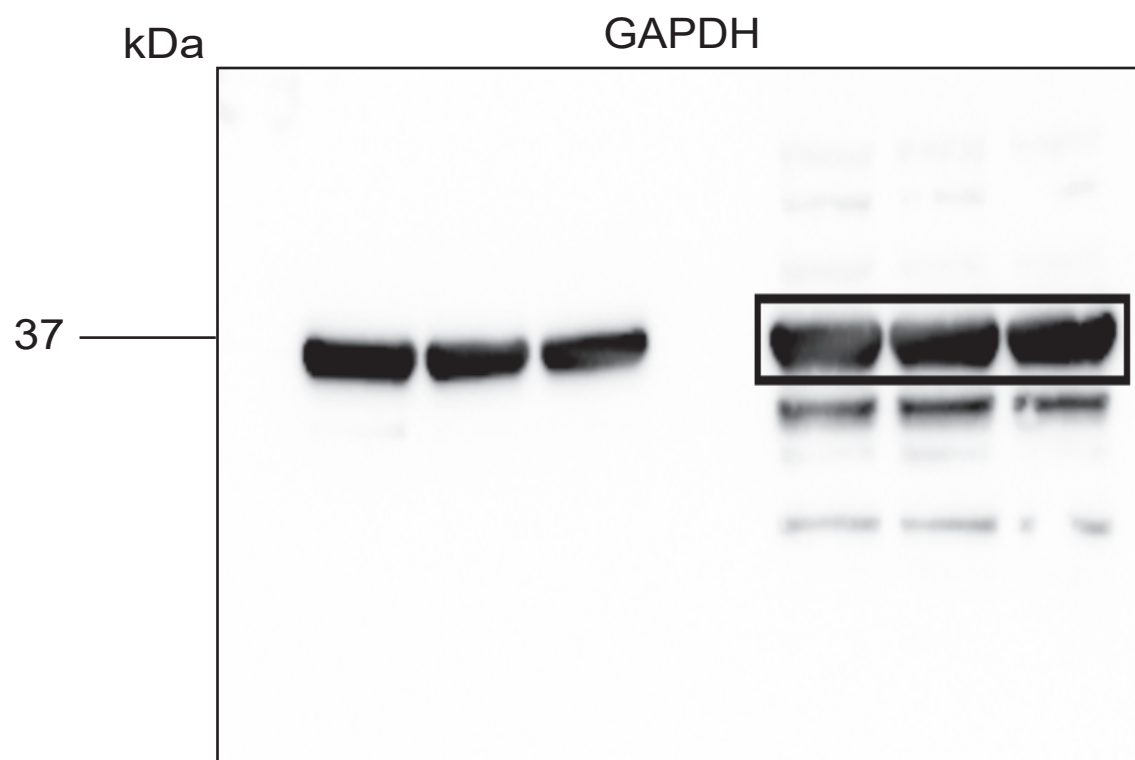

Supplement: Supplementary file 7 [file LSA-2024-03046_SdataF5.pdf]

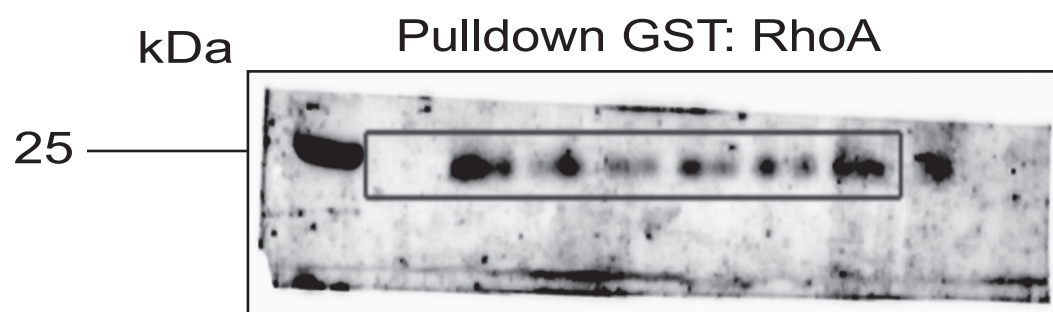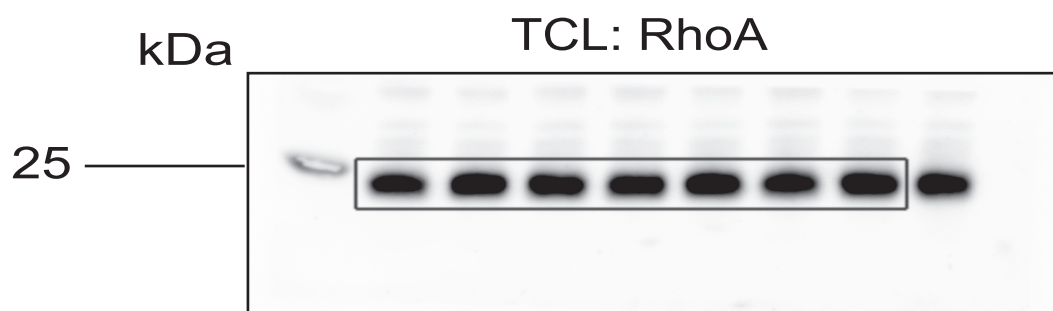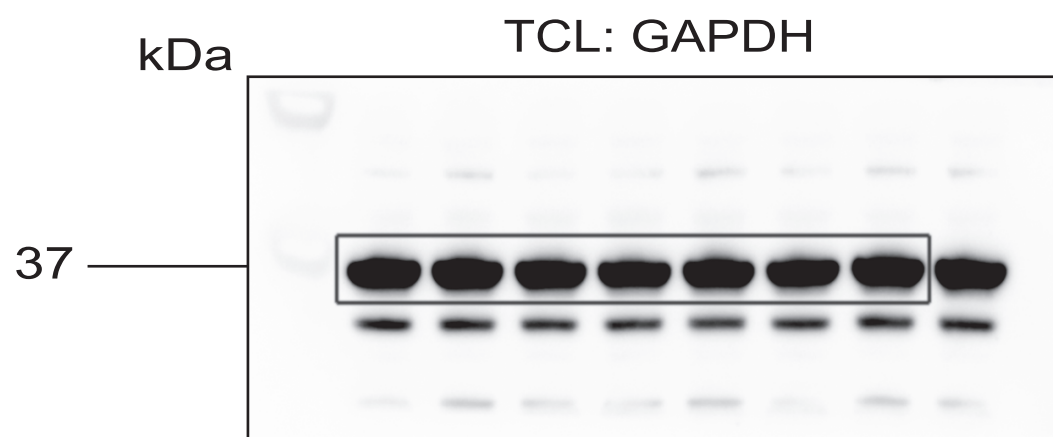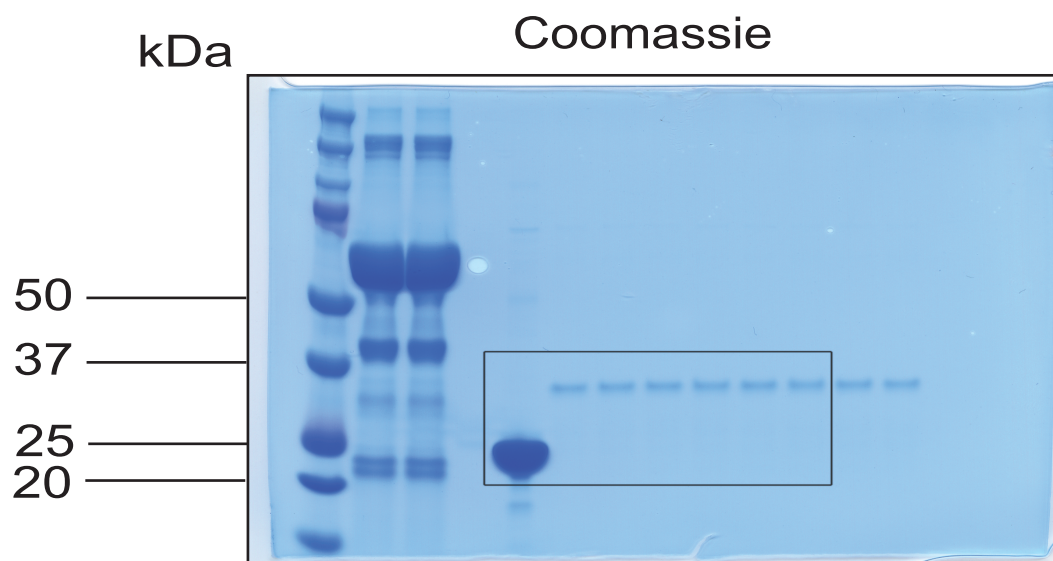

Supplement: Supplementary file 8 [file LSA-2024-03046_SdataF6.pdf]
